# Toll-Like Receptor 2 and Toll-Like Receptor 4-Dependent Activation of B Cells by a Polysaccharide from Marine Fungus *Phoma herbarum* YS4108

**DOI:** 10.1371/journal.pone.0060781

**Published:** 2013-03-29

**Authors:** Xian Zhang, Ran Ding, Yan Zhou, Rui Zhu, Wei Liu, Lei Jin, Wenbing Yao, Xiangdong Gao

**Affiliations:** State Key Laboratory of Natural Medicines, School of Life Science and Technology, China Pharmaceutical University, Nanjing, Jiangsu, P.R. China; Sudbury Regional Hospital, Canada

## Abstract

Various natural polysaccharides are capable of activating the immune system and therefore can be employed as biological response modifiers in anti-tumor therapy. We previously found a homogenous polysaccharide from the mycelium of marine fungus *Phoma herbarum* YS4108, named YCP, exhibiting strong *in vivo* antitumor ability via enhancement of the host immune responses. To further elucidate the role of YCP as a biological response modifier, the immunomoduating activities of YCP in B cells was investigated in the current study. We demonstrated that stimulation of YCP with murine splenic B cells resulted in cell proliferation and generation of IgM antibody response. Binding of YCP to B cells was a direct, saturable and reversible event and required TLR2 and TLR4 involvement. TLR2 and TLR4 defunctionalization by either antibody blocking or allele-specific mutation significantly impaired the B-cell proliferative and IgM responses to YCP. YCP interaction with TLR2 and TLR4 led to the activation of intracellular p38, ERK and JNK, as well as the translocation of transcriptional factor NF-κB into nucleus. Furthermore, specific inhibitors of p38, ERK, JNK and NF-κB could attenuate the ability of YCP to induce B cell proliferation and IgM production. Taken together, this study has indicated for the first time the immunostimulating properties of YCP on B cells through a receptor-mediated mechanism, which involves TLR2 and TLR4 and resultant activation of MAPK and NF-κB signaling pathways, thereby highlighting the role of YCP as an efficacious biological response modifier in oncologic immunotherapy.

## Introduction

In recent decades, marine-derived fungi have garnered much attention as a rich source of novel bioactive compounds due to their strong adaptability to cold, lightless and high-pressure environments in oceanic realm [1], [2]. Among marine-derived fungi, the genus *Phoma* has been proven to be a versatile producer of secondary metabolites, including nitrophthalic acid, nonenolide, terpenoid, polyketide and naphthalenone, which are potential lead compounds for the development of new phytotoxins, PAF antagonists, anti-influenza virus and antifungal drugs [3]–[7].

In our previous studies, a novel homogenous polysaccharide referred to as YCP (YCP is the acronym of Yancheng polysaccharide) was purified from the mycelium of *Phoma herbarum* YS4108 that inhabits the sediment in the Yellow Sea area around Yancheng, China. It has a backbone of α-1,4-D-glucan with a lower proportion of α-1,6-linked glucopyranosyl and glucuronic acid residues as non-reducing terminals [8]. *In vivo* antitumor experiment showed that YCP was able to significantly inhibit the growth of xenografted tumors (Heps, S180 and Lewis) without inducing any abnormality in body weight and behavior of the experimental mice. The inhibition of tumor growth by YCP was stronger than lentinan, a well-known glycan-based anticancer drug [9]. The antitumor activity of YCP was correlated to its ability to stimulate or restore the host immune responses, such as induction of cytokine production and phagocytosis by macrophages [10], [11], promotion of splenocyte proliferation [8], activation of natural killer (NK) cells and lymphokine-activated killer (LAK) cells in tumor-bearing mice, as well as reconstitution of bone marrows in myeloablated mice after radio- or chemotherapy. Given that the strategy of oncologic immunotherapy through biological response modifiers (BRMs) has been figured out to function in clinic [12], it can be expected that the polysaccharide YCP holds much promise as a novel antitumor drug with high effectiveness and low toxicity.

Although the mechanisms underlying the immunomodulating activity of polysaccharides need to be further explored, one of the primary mechanisms involves Toll-like receptors (TLRs). The mammalian TLR family is a group of germ-line encoded receptors that trigger immune responses via recognition of structures conserved among microbial species known as pathogen-associated molecular patterns (PAMPs), such as LPS, peptidoglycan, lipoprotein, flagellin and double-stranded RNA [13], [14]. The family comprises at least 11 members, among which TLR2 and TLR4 are well characterized as the transmembrane receptors involved in the recognition of ligands containing carbohydrate moieties, e.g. peptidoglycans [15], lipopolysaccharide (LPS) [16] and various natural polysaccharides [17]–[19]. Upon sensing the presence of these ligands, TLRs trigger the downstream signaling cascade of MyD88/TIRAP-IRAK1-TRAF6-TAK1, which, in turn, results in the activation of mitogen-activated protein kinases (MAPKs) and nuclear factor -κB (NF-κB), and further leads to the regulation of genes that orchestrate the proliferation, survival and immune responses [20], [21].

In this study, we investigate the immunomodulating property of YCP in murine splenic B cells, especially focusing on the involvement of TLR signaling in YCP-mediated B cell responses. We find that YCP is capable of inducing proliferation and antibody production in B cells, the mechanism of which is direct, saturable and reversible binding of YCP to TLR2 and TLR4 with subsequent activation of MAPK and NF-κB signaling pathways. Collectively, these data show that YCP is an efficacious stimulant of B cell function.

## Results

### YCP promotes B cell proliferation and induces IgM response

**Figure 1:**
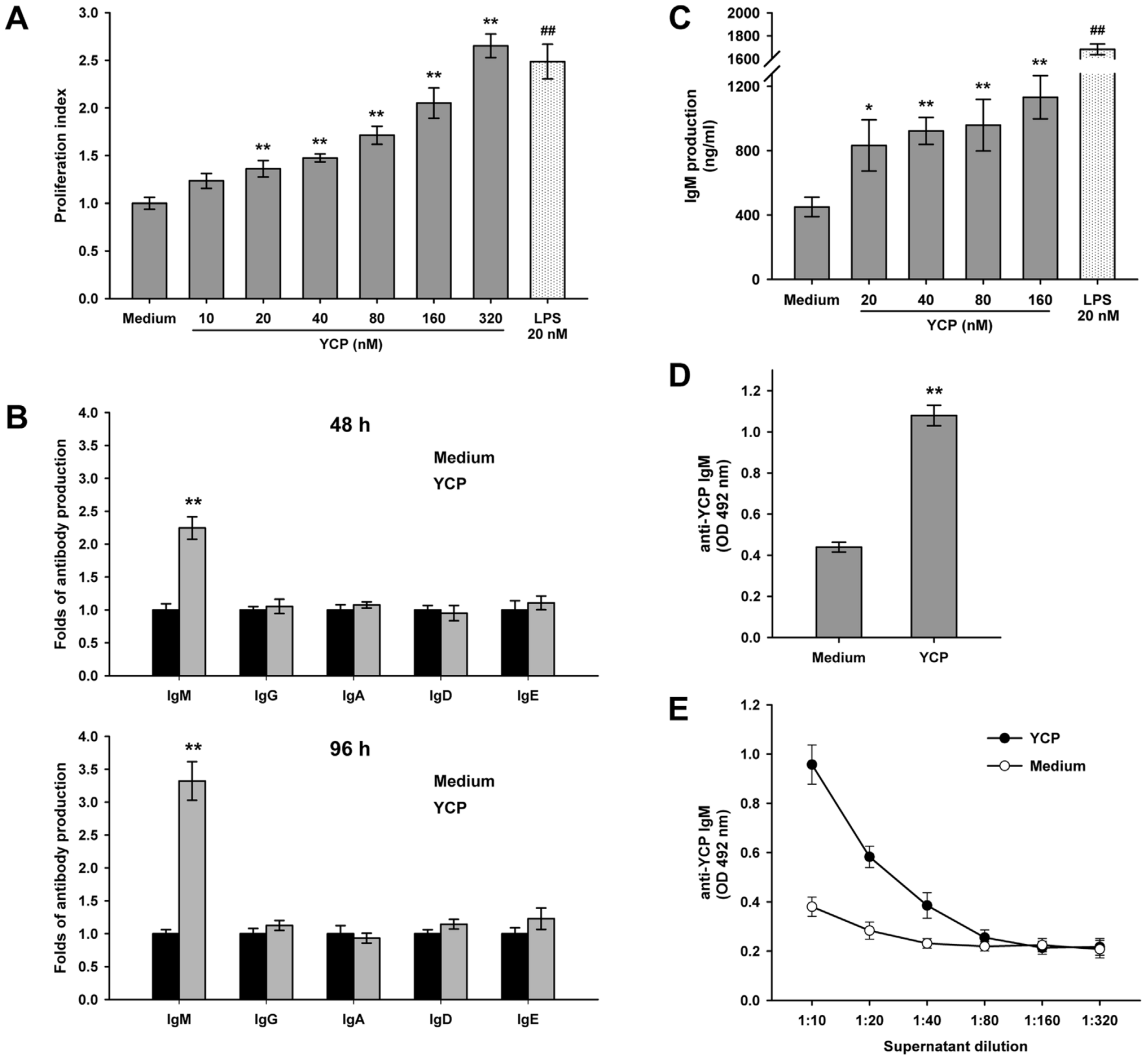
YCP promotes B cell proliferation and induces IgM response. **A:** Effect of YCP on B cell proliferation. Splenic B cells were treated with YCP or LPS at the indicated concentrations, and cell proliferation was measured by MTT assay at 48 h (n = 6, ^**^ *p*≤0.01 vs. medium by Kruskal–Wallis test followed by Dunn's post-hoc test, ^##^ *p*≤0.01 vs. medium by Mann–Whitney U test). **B:** The production of IgM, IgG, IgA, IgD and IgE in response to YCP stimulation. Splenic B cells were incubated with 80 nM of YCP for 48 h or 96 h, and supernatants were then collected for quantification of each Ig isotype using sandwich ELISA (n = 5, ^**^ *p*≤0.01 vs. medium by Mann–Whitney U test). **C:** A dose-dependent induction of IgM antibodies by YCP at 48 h (n = 6, ^*^ *p*≤0.05, ^**^ *p*≤0.01 vs. medium by Kruskal–Wallis test followed by Dunn's post-hoc test, ^##^ *p*≤0.01 vs. medium by Mann–Whitney U test). **D:** IgM reactivity to YCP in supernatants from naïve and YCP-treated B cells. Splenic B cells were left untreated or treated with YCP for 48 h, and the supernatants collected from each group were subjected to measurement of YCP-bound IgM using indirect ELISA (n = 6, ^**^ *p*≤0.01 vs. medium by Mann–Whitney U test). **E:** Titration of IgM antibodies to YCP in supernatants from naïve and YCP-stimulated B cells (n = 6, ^**^ *p*≤0.01 vs. medium by Mann–Whitney U test).

Splenic B cells were isolated by nylon fibers to a purity ≥90% (data not shown), and then cultured with YCP or LPS, a well-known B cell stimulant, as the positive control. Cell proliferation was measured after 48 h by MTT assay. The results indicated that YCP significantly stimulated B cell to proliferate in a dose-dependent manner (**Figure 1A**). The stimulation obtained with YCP was weaker than that mediated by equimolar amounts of LPS, suggesting YCP may be a safe and effective immunostimulant which doesn't induce an acute and robust inflammation as LPS.

In addition to the proliferative effect, the effect of YCP on antibody production from B cells was evaluated. To find YCP mainly elicit B cell response through which isotype of antibodies, the production of IgM, IgG, IgA, IgD and IgE in response to YCP stimulation was investigated, respectively, at 48 or 96 h. Among all isotypes only IgM levels showed a significant increase after YCP treatment for 48 h. A similar result was also obtained at 96 h, which indicated that YCP promoted the production of IgM more potently when compared with the other isotypes (**Figure 1B**). A dose-dependent induction of IgM by YCP at 48 h was shown in **Figure 1C**. To further measure the reactivity of IgM, the supernatants collected from naïve and YCP-stimulated cells were tested for its binding ability to YCP by ELISA. IgM antibodies elicited by YCP-stimulated cells were more reactive to YCP than those secreted by naïve cells (**Figure 1D**). To rule out a prozone effect, supernatants were sequentially diluted by twofold and then subjected to measurement of anti-YCP IgM. Only marginal level of anti-YCP IgM was detected in naïve B cells at any dilution (**Figure 1E**). Since the difference in total IgM level between naïve and YCP-stimulated cells has been normalized prior to reactivity analysis, it could be expected that the induction of anti-YCP IgM was antigen-driven, not completely due to polyclonal B cell expansion.

Taken together, we proposed that the polysaccharide YCP was an efficacious B cell stimulant capable of promoting cell proliferation and eliciting primarily IgM antibody responses.

### YCP binds to B cells directly, saturably and reversibly

**Figure 2:**
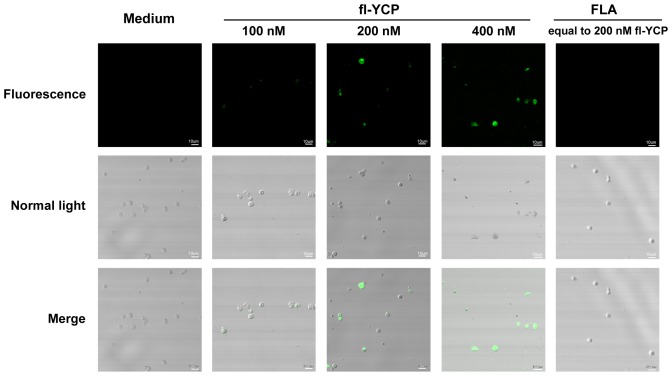
Fluoresceinamine-labeled YCP binds to B cells positively. Splenic B cells were incubated with media alone, fl-YCP, or FLA at the indicated concentrations for 1 h at 4°C, and then spotted on a slide and subjected to confocal laser scanning. Experiments were performed in triplicate. The representative of cells with fluorescence and normal light observation and a merged image for each group are presented.

It is known that many polysaccharides can exert their immunomodulating activities via directly binding to specific receptors or partners on immunocytes. To investigate whether YCP stimulated B cells through the same mechanisms, we prepared fluoresceinamine-labeled probe of YCP (fl-YCP) and examined its direct binding capacity to B cells by confocal microscope analysis and flow cytometry. It was observed clearly that B cells incubated with fl-YCP showed bright fluorescence under the confocal microscope whereas the control group exhibited no fluorescence, suggesting the positive binding of fl-YCP to B cells. The FLA moiety-dependent binding of fl-YCP was excluded by the faint fluorescence found in cells treated with FLA at an equimolar dose (**Figure 2**).

**Figure 3:**
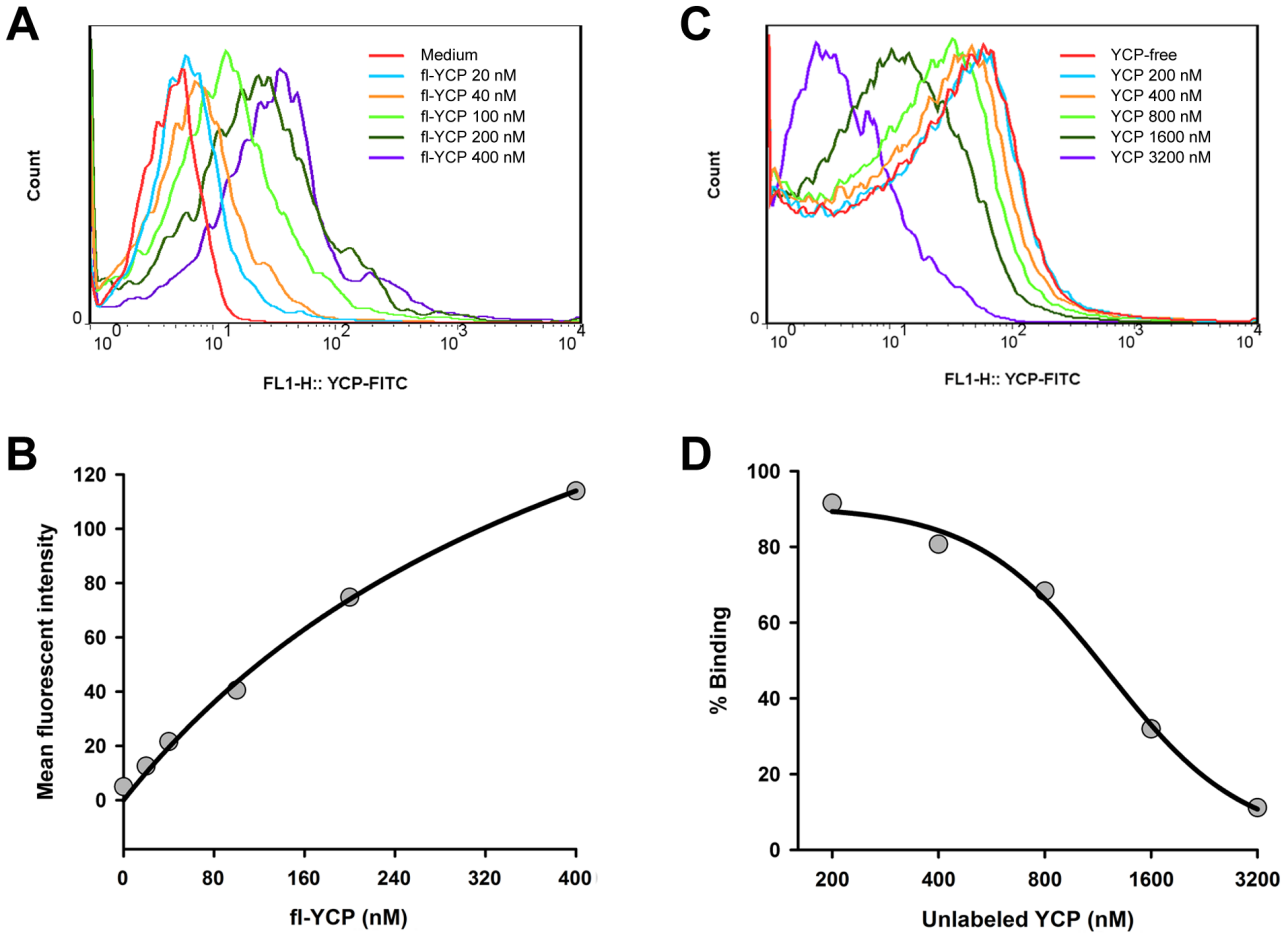
YCP binds to B cells in a saturable and reversible manner. **A** and **B:** Saturable binding kinetics of YCP in B cells. Splenic B cells were incubated with fl-YCP at the indicated concentrations for 1 h at 4°C. The mean fluorescence intensity of each group was examined by flow cytometry. The histogram overlays shown in panel A are representative of triplicates, and the combined results are presented in panel B. **C** and **D:** Competitive inhibition of fl-YCP binding to B cells by unlabeled YCP. Cells were incubated with 100 nM of fl-YCP for 1 h in the absence or presence of unlabeled YCP at concentrations from 200 to 3 200 nM, and then harvested for flow cytometric analysis. The histogram overlays shown in panel C are representative of triplicates, and the combined results expressed as a percentage mean fluorescence intensity of fl-YCP with no unlabeled YCP are presented in panel D.

Next, both saturation and competition assay were performed to study the binding characteristics of fl-YCP in B cells. In saturation assay, B cells were stained with fl-YCP at serial concentrations for 1 h, and then determined for the binding ability of fl-YCP by flow cytometry. As shown in **Figure 3A and B**, the mean fluorescent intensity of B cells was gradually elevated with the incremental increases in fl-YCP concentration ranging from 20 to 400 nM, and reached the saturation state at higher doses. The *K*_d_ and *B*_max_ value for YCP binding to B cells were ∼ 490 nM and 248, respectively. In competition assay, B cells were incubated with fl-YCP and excess unlabeled YCP together for 1 h and then subjected to flow cytometric analysis. As expected, additional unlabeled YCP from 200 to 3 200 nM (from 2- to 16-fold of fl-YCP) resulted in a dose-dependent decrease in the mean fluorescent intensity of B cells, suggesting fl-YCP binding to B cells can be competitively inhibited by unlabeled YCP. Curve-fitting analysis by 4-parameter logistic model showed that YCP had an IC_50_ of ∼ 1300 nM (**Figure 3C and D**). All of these observations indicated that YCP binding to B cells was a direct, dose-dependent, saturable and reversible process, and implied that the stimulation of B cells by YCP may be a receptor-mediated event.

### TLR2 and TLR4 are required for YCP activities in B cells

TLRs have been demonstrated to play a direct role in control of B cell responses including proliferation, up-regulation of activation markers, cytokine secretion, terminal differentiation and antibody production [22]–[24]. Based on this finding, and taking into consideration that TLR2 and TLR4 can recognize carbohydrate-containing molecules, we focused our study on the functional relevance of TLR2 and TLR4 in YCP-mediated B cell responses.

**Figure 4:**
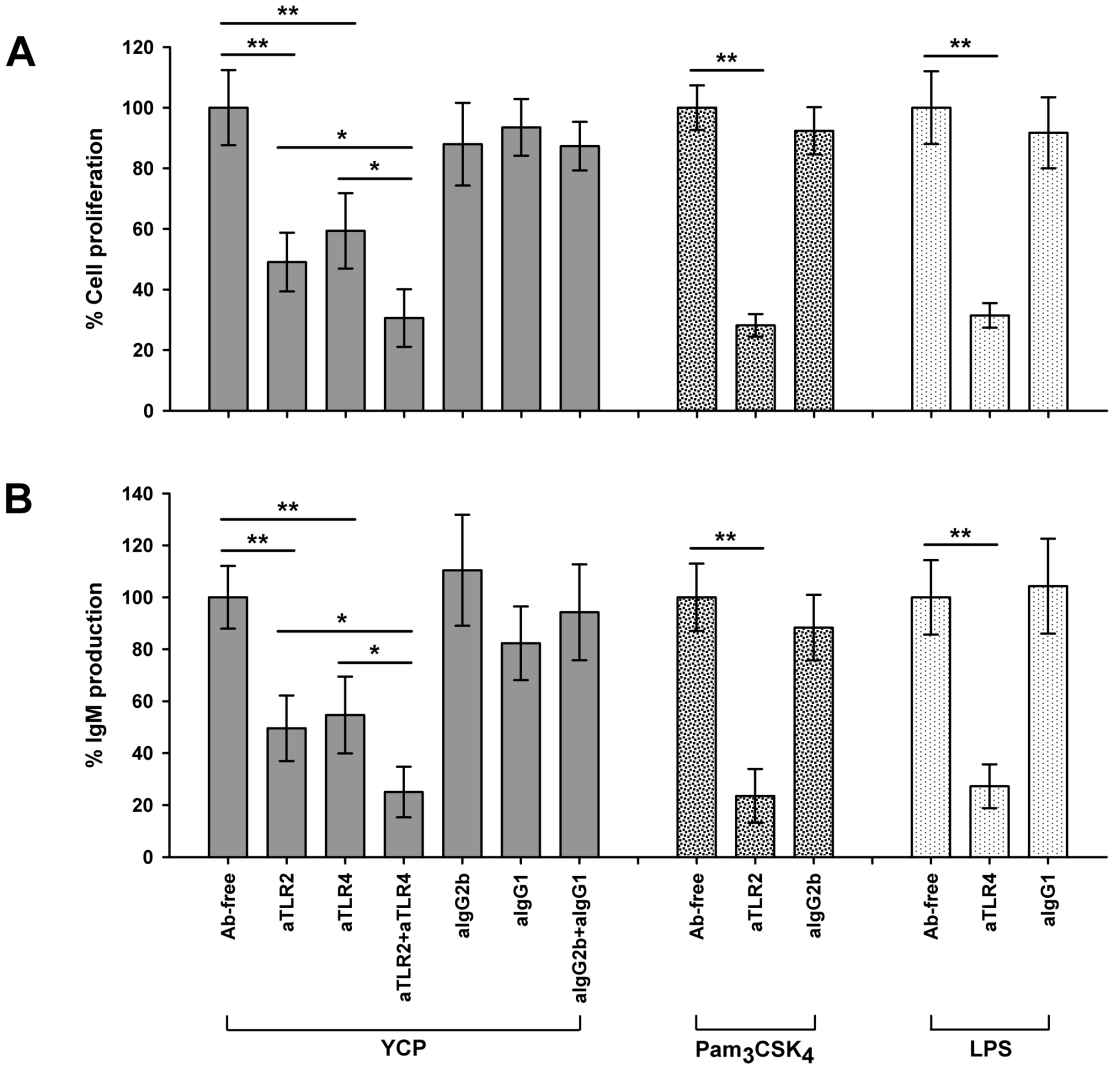
Antibodies to TLR2 and TLR4 attenuate YCP functions in B cells. Splenic B cells were pretreated with anti-TLR2, anti-TLR4 or respective isotype controls (20 µg/ml) for 2 h before the addition of YCP. After 48 h incubation, cell proliferation (A) and total IgM concentration in supernatants (B) were measured using MTT assay or ELISA, respectively (n = 6, ^*^ *p*≤0.05, ^**^ *p*≤0.01 by Mann–Whitney U test).

The requirement of TLR2 and TLR4 for YCP function was first investigated in B cells following receptor blocking with specific antibodies. B cells were stimulated with YCP in the presence of antibodies to TLR2 or TLR4, and then subjected to proliferation assay and total IgM quantification. Antibodies to TLR2 and TLR4 significantly reduced the proliferative effect of YCP by 50.9% and 40.6%, respectively, when compared with Ab-free control (**Figure 4A**), and suppressed the induction of IgM production by 50.4% and 45.3% in each case (**Figure 4B**). The reduction was found to be synergetic upon the combined treatment with anti-TLR2 and anti-TLR4. To eliminate the possibility of a gross defect in responsiveness of cells as a consequence of addition of antibodies, B cells pretreated with nonspecific Ig isotypes (IgG2b or IgG1) were taken as the negative control. In contrast to the substantial suppression observed in anti-TLR2 and/or anti-TLR4 treatment group, neither proliferation nor IgM production was significantly altered in YCP-stimulated B cells in the presence of isotype control (**Figure 4A and B**). Therefore, we proposed that TLR2 and TLR4 might be functionally correlated to the stimulation of B cells by YCP.

**Figure 5:**
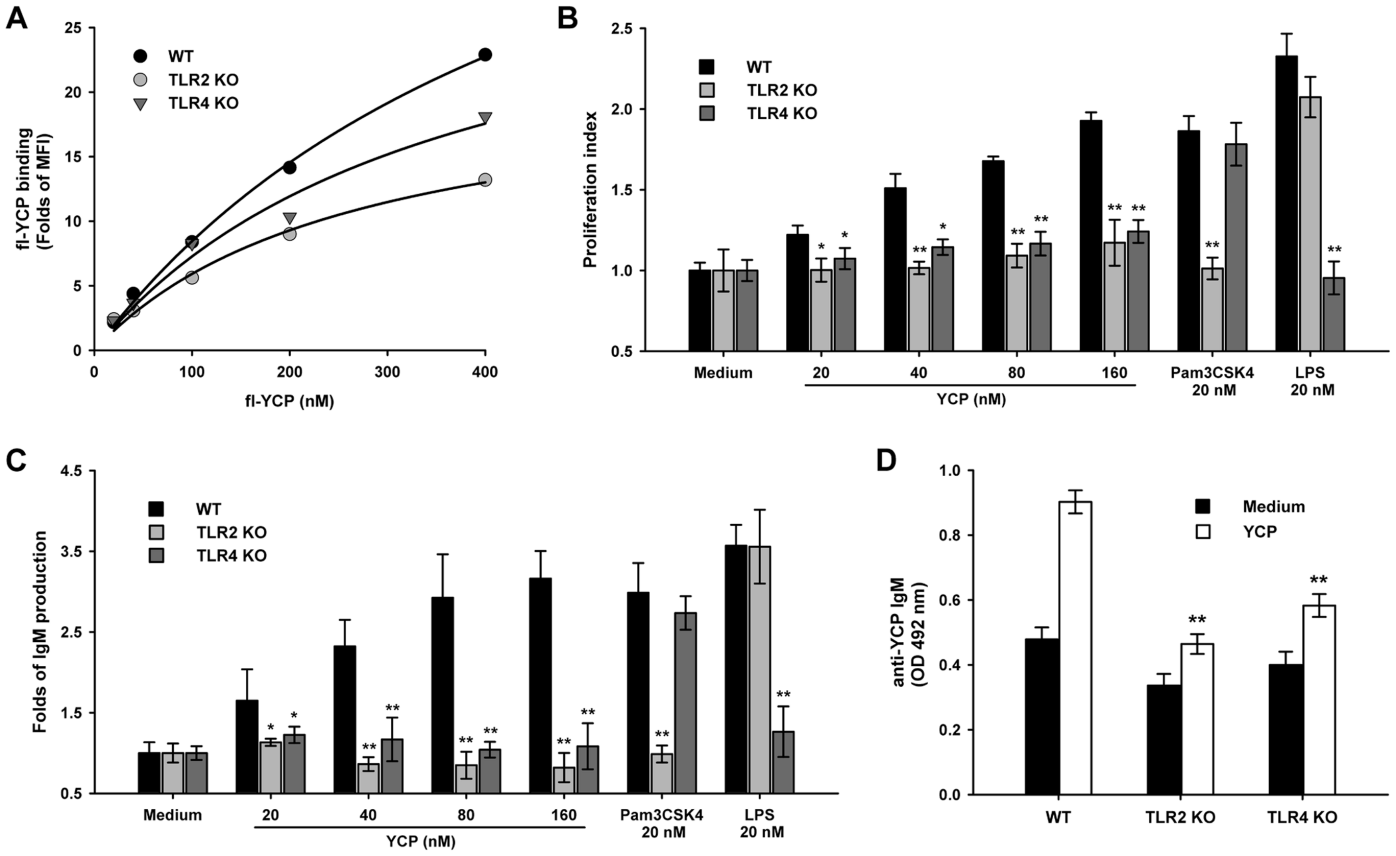
YCP possesses a lower binding affinity and mediates a hyporesponsiveness in TLR2 or TLR4 KO B cells. **A:** Splenic B cells from WT (BALB/c), TLR2 KO (B6.129-Tlr2^tm1Kir/J^ and TLR4 KO (C57BL/10ScNJ) mice were incubated with fl-YCP for 1 h, and then harvested for measurement of mean fluorescence intensity (MFI) by flow cytometry. The experiments were performed in triplicate, and the combined results are presented. **B–D:** WT, TLR2 and TLR4 KO B cells were stimulated with YCP, Pam_3_CSK_4_ or LPS for 48 h, and then subjected to proliferation assay (B) and total IgM quantification (C). The supernatants obtained from each untreated or YCP-treated group were assayed for the content of anti-YCP IgM by indirect ELISA (D) (n = 6, ^*^ *p*≤0.05, ^**^ *p*≤0.01 vs. WT by Mann–Whitney U test).

To further confirm this statement, the impact that loss of TLR2 or TLR4 has upon YCP activities was evaluated in B cells from either B6.129-Tlr2^tm1Kir/J^ (TLR2 KO) or C57BL/10ScNJ (TLR4 KO) mice. B cells deficient in TLR2 or TLR4 had lower *K*_d_ and *B*_max_ for YCP binding when compared to the wide-type B cells from BALB/c mice (**Figure 5A**). The proliferation and IgM production in response to YCP in TLR2/4 KO B cells were also less potent when compared to those in wide-type B cells. The deficiency in TLR2/4 function was specific, since knocking-out of TLR2 allele from the genome only impaired their B-cell proliferative and antibody response to Pam_3_CSK_4_ (a bona fide TLR2 agonist) rather than LPS (a bona fide TLR4 agonist), and vice versa (**Figure 5B and C**). Furthermore, a lower level of anti-YCP IgM was detected in the supernatants from TLR2/4 KO B cells (**Figure 5D**). These findings, together with those from antibody blocking assay, provided convincing evidence that both TLR2 and TLR4 were responsible for the immunostimulating property of YCP in murine splenic B cells.

### MAPK signaling is greatly involved in YCP-induced B cell response

**Figure 6:**
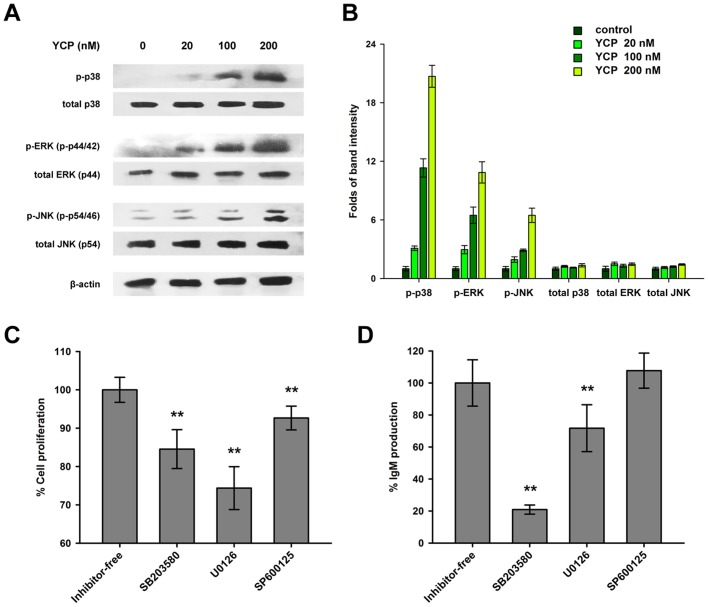
YCP-induced B cell activation involves MAPK signaling. **A** and **B:** YCP-mediated MAPK activation in B cells. Splenic B cells were treated with YCP at the indicated concentrations for 30 min, and then cellular extracts obtained from each group were subjected to Western blot analysis probing with anti-p38, anti-phospho-p38, anti-ERK, anti-phospho-ERK, anti-JNK, anti-phospho-JNK, or β-actin antibody. Images shown in panel A are representative of triplicates, and the band intensities are presented in panel B. **C** and **D:** Abrogation of YCP-mediated B cell proliferation and IgM production by specific MAPK inhibitors. Cells were stimulated with YCP for 48 h along with a pretreatment with either SB203580, U0126 or SP600125 for 30 min. Cell proliferation (C) and IgM production in supernatants (D) were measured using MTT assay or ELISA, respectively (n = 6, ^**^ *p*≤0.01 vs. inhibitor-free by Mann–Whitney U test).

To evaluate the consequential downstream events upon YCP binding to TLR2 and TLR4 in B cells, the responses of three MAPK proteins including p38, ERK and JNK, which have been well-characterized as the signal transducers for TLR2 and TLR4, were investigated [20]. To start with, B cells were treated with different concentrations of YCP (20, 100, 200 nM) for 30 min. Then the cellular extracts from each group were subjected to the detection of MAPK phosphorylation by Western blot analysis. As shown in **Figure 6A and B**, YCP increased the phosphorylation of p38 (at Tyr182), ERK (at Tyr204) and JNK (at Thr183 and Tyr185) in a dose-dependent manner, without affecting the total expression of p38, ERK or JNK significantly. These findings suggested that YCP can trigger all of the three MAPK signaling pathways in B cells.

To further elucidate the relevance of the MAPK pathways to YCP activities in B cells, specific inhibitors of p38 (SB203580), ERK (U0126) and JNK (SP600125) were used to examine if these inhibitors could interfere with YCP-mediated B cell responses. We found that 20 µM of p38 inhibitor SB203580 decreased YCP-dependent cell proliferation and IgM production by 15.5% and 79.1%, respectively, when compared with inhibitor-free control, suggesting that p38 MAPK signaling, at least partly, played a pivotal role during the B cell activation process (**Figure 6C and D**). A similar effect can also be observed from the experiment performed with the ERK inhibitor U0126 in which additional usage of U0126 at 20 µM resulted in 25.6% suppression in YCP-mediated cell proliferation and 28.3% suppression in IgM production (**Figure 6C and D**). Unlike SB203580 and U0126, the JNK inhibitor SP600125 at an equimolar dose only reduced the proliferative activity of YCP in B cells by 7.4%, while had no inhibitory effect on the induction of IgM production (**Figure 6C and D**). Combining the data from signaling inhibition assay and Western blot analysis, we found that YCP induced IgM production through p38 and ERK signaling in B cells, while a broader profile of signaling pathways including p38, ERK and JNK was utilized by YCP to promote B cell proliferation.

### YCP stimulation leads to the activation of NF-κB in B cells

**Figure 7:**
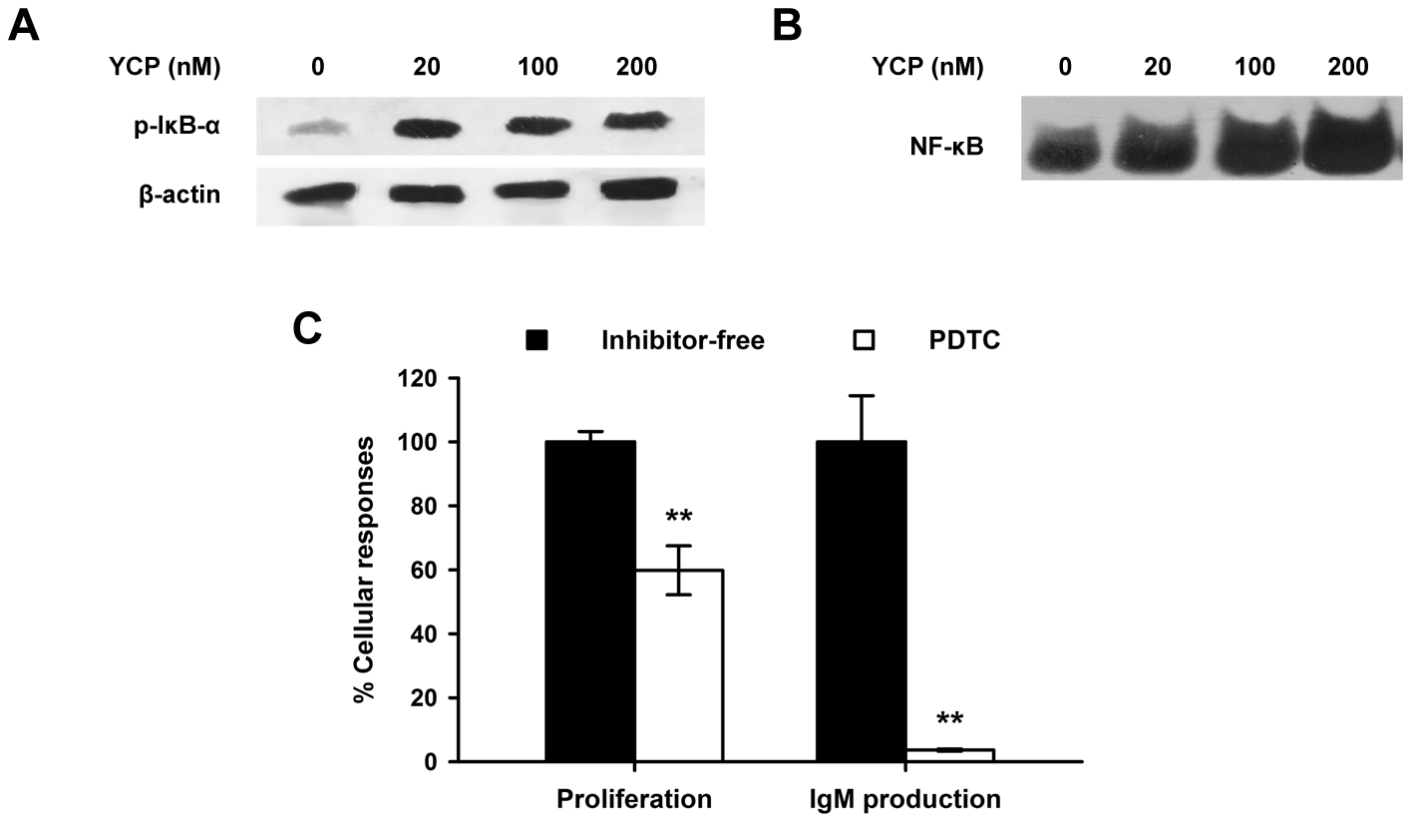
YCP stimulation leads to the activation of NF-κB in B cells. **A:** YCP-mediated IκB-α phosphorylation in B cells. Splenic B cells were treated with YCP at the indicated concentrations for 1 h, and cellular extracts were prepared for Western blotting analysis with anti-phospho-IκB-α. Images shown here are representative of triplicates. **B:** Effect of YCP on NF-κB binding activity. Splenic B cells were treated with YCP at the indicated concentrations. After 1 h, the nuclear extracts obtained from each group were analyzed by EMSA using the biotin-labeled probe containing binding sequence for NF-κB. Images shown here are representative of triplicates. **C:** Abrogation of YCP-mediated B cell proliferation and IgM production by specific NF-κB inhibitor. Cells were treated with PDTC for 30 min prior to stimulation with YCP for 48 h. Cell proliferation and IgM production in supernatants were measured using MTT assay or ELISA, respectively (n = 6, ^**^ *p*≤0.01 vs. inhibitor-free by Mann–Whitney U test).

Previous studies have shown that signaling through TLRs may contribute to NF-κB activation by inducing the phosphorylation and degradation of IκB and the translocation of NF-κB into nucleus [21], [25]. In view of the ability of YCP to initiate TLR2 and TLR4 signaling in B cells, we asked whether YCP stimulation can consequently lead to the activation of NF-κB by measuring the phosphorylation of IκB-α using Western blot analysis. At all indicated doses (20, 100, 200 nM), YCP dramatically elevated the level of IκB-α phosphorylation compared with untreated control, although no significant changes were observed among the cells treated with different doses of YCP (**Figure 7A**). To further study the effect of YCP on NF-κB activation, the binding of NF-κB to DNA as a downstream event of IκB-α phosphorylation was determined by EMSA. As presented in **Figure 7B**, only a low level of NF-κB/DNA complex can be found in untreated B cells, whereas a large quantity of complex was detected in YCP-treated cells, suggesting that the binding activity of NF-κB to DNA was significantly up-regulated by the treatment of YCP. Furthermore, the specific NF-κB inhibitor PDTC (20 µM) inhibited the proliferative effect of YCP by 40.2% and almost completely abrogate the induction of IgM production, which indicated the functional relevance of NF-κB to YCP activities in B cells (**Figure 7C**). Altogether, these findings led to the conclusion that YCP is able to trigger NF-κB signaling in B cells, which is essential for YCP-dependent B cell activation.

## Discussion

Immunologically, an antigen can be classified either as T cell dependent (TD) or T cell independent (TI). Most of polysaccharides have been considered as classic TI antigens. They are inclined to elicit B-cell immune responses comprising low affinity IgM production, restricted IgG class switching, and no immunologic memory [26], [27]. The classification of polysaccharides as TI antigens is based on the lack of T cell dependent responses to these typically negatively charged or uncharged molecules [28]. In agreement with this, our current study indicated that the polysaccharide YCP was capable of inducing primarily IgM response in purified murine B cells. The inducible IgM antibodies were reactive to YCP with a relatively low titer (<40). The characteristics of YCP as a TI antigen observed *in vitro* was highly consistent with our findings from the immunized mice showing that immunization with the YCP induced low or undetectable levels of YCP-specific IgM (mean titer≤32) or IgG (mean titer<8) antibodies in mice after primary immunization and this was not increased following subsequent immunization. By contrast, conjugation of YCP to a carrier protein, e.g. BSA, enabled YCP to elicit a TD antibody response typified by the substantial production of high-affinity IgG to YCP and the induction of immunologic memory [29].

The engagement of TLRs in the induction of B-cell response has been studied during the last decade. For example, most TLR agonists, such as Pam_3_CSK_4_ (TLR1/2), MALP-2 (TLR2/6), LPS (TLR4), R848 (TLR7), and CpG-DNA (TLR9), are demonstrated to promote proliferation of all B cell subsets, and lead to massive differentiation of marginal zone (MZ) and B-1 subsets into mature plasma cells without involvement of B cell receptor (BCR) [30]. In addition to the BCR-independent pathway, TLRs have also been shown to synergize with BCR-mediated signals to generate the antigen-specific responses [31]–[33]. Based on this knowledge, and taking consideration of the TLR2/4 ligand nature of YCP, we proposed that YCP may elicit B-cell response through the following two mechanisms. Firstly, YCP stimulates MZ and B-1 B cell differentiation and polyclonal B cell expansion, both of which result in enhancement of natural IgM antibody production [34]. Natural antibodies represent a collection of germ-line-encoded antigen-recognition molecules, which is polyreactive to a conserved pattern in many pathogens. Due to this polyreactivity and the higher sensitivity of MZ and B-1 B cells to stimulus, the natural antibodies are able to neutralize pathogens rapidly prior to the generation of high-affinity and specific antibodies, thereby contributing to the innate host defense [35], [36]. Secondly, since YCP as a polymer possesses highly repetitive structure, it could be regarded as a TI type 2 (TI-2) antigen which induces B-cell activation and proliferation by extensive cross-linking of antigen-bound membrane Ig (mIg) molecules. While this first signal of multivalent mIg cross-linking can induce B-cell proliferation, a second signal, which has been characterized as TLR signaling, is required for a TI-2 Ig secretory response [22], [37]. Our data were in agreement with this showing that B cells expressing non-functional TLR2/4 produced anti-YCP IgM at a much lower degree as compared to WT cells (**Figure 5D**). Taken together, it may suggest that the multiple repeating antigenic epitopes of YCP as well as the TLR-recognized molecular motifs on YCP surface could trigger a dual BCR/TLR signaling to mediate YCP-specific IgM response.

Although various carbohydrate-containing macromolecules have been demonstrated to be TLR2 and TLR4 ligands as stated above and elsewhere, no or only few significant structural similarities could be found among most of them despite sharing an overlapping receptor profile and immunomodulating activity. For instance, YCP is an α-1,4 linked glucan with α-1,6-branched glucose and glucuronic acid [8], whereas another TLR2 and TLR4 ligand, glucuronoxylomannans, is composed by α-1,3 linked mannans that present substitutions in O-acetylated mannosyl residues with β-1,2 glucuronyl and β-1,2/β-1,4 xylosyl units [38]. On the other hand, some structurally similar polysaccharides have been found to differ in their receptor profiles. A potent example of this is ESG, an α-1,6-branched α-1,4-glucan, activates immunocytes through TLR2 but not TLR4 although it has analogous monosaccharide composition and glycosidic linkages with YCP [39]. Also, another α-1,4-D-glucan purified from *Tinospora cordifolia* was shown to exert its immunostimulating activitiy with a mechanism that involved TLR6 but not TLR2 and TLR4 [40]. Therefore, we proposed that the polysaccharide ligands may be recognized by their cognate receptors more dependently on the three-dimensional conformations than the fine sugar chain structures. The substantial overlap in the receptors and immunomodulating properties of natural polysaccharides in the face of considerable structure diversity might be explained if these polysaccharides assume similar three-dimensional conformations and define a common scaffold to form a unique molecular pattern for reorganization [41]. The newly reported crystal structures of TLR2-TLR1 and TLR4-MD2 complex with their respective ligands, Pam_3_CSK_4_ and LPS, have suggested that the lipid chains in Pam_3_CSK_4_ and LPS can be inserted into the large hydrophobic pockets in TLR complex to mediate m-shaped receptor multimer formation, and thus may be the conserved molecular pattern recognized by TLR2 and TLR4 [42], [43]; however, since YCP is completely composed by hydrophilic carbohydrates without any lipid chains, the way of TLR2-YCP and TLR4-YCP interaction or, in other words, the structures essential for YCP forming a molecular pattern remain to be resolved in our future studies.

In summary, we have demonstrated the immunomodulating potential of a marine-derived polysaccharide YCP in murine B cells. The most likely mechanism accounting for this involves TLR2- and TLR4-mediated MAPK and NF-κB signaling pathways. Given that immunotherapy has become an important component of cancer treatment during recent decades, our findings clearly support the role of YCP as an efficacious and safe biological response modifier which might be applied in the future clinical practice and antitumor therapy.

## Materials and Methods

### Ethics Statement

All experimental procedures with animals used in the present study were in accordance with the Guide for the Care and Use of Laboratory Animals as adopted and promulgated by the United States National Institutes of Health, and had been given prior approval by Jiangsu Provincial Experimental Animal Manage Committee under Contract 2007(su)-0025.

### Reagents

RPMI-1640 medium and FCS were obtained from Gibco (Grand Island, NY, USA). ELISA kits for measuring murine IgA, IgD, IgE and IgG were purchased from R&D Systems (Minneapolis, MN, USA) and that for measuring IgM was from Mabtech (Nacka Strand, Sweden). Rat anti-mouse IgM and peroxidase-conjugated anti-rat IgG were from Biolegend (San Diego, CA, USA). Abs specific to mouse TLR2 (rat IgG2b isotype), TLR4 (mouse IgG1 isotype) and the isotype controls were from eBioscience (San Diego, CA, USA). Abs to p38, ERK, JNK, phospho-p38, phospho-ERK, phospho-JNK, phospho-IκB-α and β-actin were all from Santa Cluz (Santa Cluz, CA, USA). SB203580, U0126, SP600125, PDTC, biotin-labeled NF-κB probe and BCA protein assay kit were from Beyotime (Nantong, Jiangsu, China). Chemiluminescent EMSA kit and ECL Western blotting substrate were from Pierce (Rockford, IL, USA). Pam_3_CSK_4_ and protease/phosphatase inhibitor cocktails were from Biovision (Mountain View, CA, USA). LPS, 3-(4,5-dimethylthiazol-2-yl)-2,5-diphenyltetrazolium bromide (MTT), o-phenylenediamine, fluoresceinamine (FLA) and 1-cyano-4-dimethylaminopyridine tetrafluoroborate (CDAP) were from Sigma-Aldrich (St. Louis, MO, USA).

### Animals

BALB/c mice between 6 and 8 weeks old were provided by the Experimental Animal Center of Yangzhou University (Yangzhou, Jiangsu, China). B6.129-Tlr2^tm1Kir/J^ (TLR2 KO) and C57BL/10ScNJ (TLR4 KO) mice between 6 and 8 weeks old were provided by the Model Animal Research Center of Nanjing University (Nanjing, Jiangsu, China). All mice were housed in a specific pathogen-free (SPF) facility under normal laboratory conditions, i.e., room temperature (22±2°C), 45–55% relative humidity, and 12/12 h light–dark cycle with free access to standard rodent chow and water.

### B cell isolation

Murine splenic B cells were prepared according to the method described previously [44] with slight modification. Briefly, spleens were gently homogenized in RPMI-1640 medium. Erythrocytes were removed by hypotonic lysis using NH_4_Cl and adherent cells were separated by plating at 37°C for 4 h. The non-adherent cells were collected as splenocytes and applied to a nylon fiber column (Wako, Osaka, Japan). T cells were gently eluted without adding any additional pressure and B cells were flushed out of the column using a plunger. The purity of B cells was characterized through FACS analysis for CD19 expression; the ratio of CD19^+^ cells in B cell fraction was approximately 90%.

### Purification of polysaccharide YCP

The polysaccharide YCP was prepared from the mycelium of marine filamentous fungus *Phoma herbarum* YS4108 following the protocols described previously [8]. The purity of YCP was shown as a single chromatographic peak on an Agilent 1100 HPLC system equipped with a gel permeation chromatographic column Shodex KS-805 (Showa Denko K.K., Tokyo, Japan) (**Figure S1A**). The polydispersity of YCP was estimated to be approximately 1 (D = 1.06), indicating YCP possesses a good homogeneity in molecular weight (**Figure S1B**). In addition, no absorption at 280 or 260 nm in the UV spectrum (**Figure S1C**) and undetectable contents of N and P by element analysis (**Figure S1D**) suggested the absence of proteins or nucleic acids in YCP sample. The endotoxin contamination in YCP was also proved to be negligible by LAL chromogenic endpoint assay (data not shown).

### Proliferation assay

B cells were cultured in 96-well microplates at a density of 2×10^6^ cells/ml in RPMI-1640 medium containing 10% FCS, supplemented with 60 mg/l penicillin, 100 mg/l streptomycin. The cells were stimulated with YCP (20–320 nM), LPS (20 nM) or Pam_3_CSK_4_ (20 nM) for 48 h in a CO_2_ incubator, followed by incubation with MTT (5 mg/ml) for another 4 h. The formazan crystals formed from MTT by living cells were fully dissolved in DMSO for 10 min. The absorbance was determined at 570 nm in a multiskan spectrum (Thermo Fisher Scientific, Vantaa, Finland), and induction of cell proliferation was expressed as the proliferation index, calculated by dividing *A*_570_ of stimulated cells with *A*_570_ of control cells [44].

### ELISA

B cells were seeded onto 96-well microplates at a density of 2×10^6^ cells/ml and incubated with YCP (80 nM) at 37°C for 48 h. Cell-free supernatants were collected for quantification of total IgM, IgG, IgA, IgD, and IgE levels by commercial ELISA kits according to the manufacturer's protocols.

The culture supernatants derived from medium- or YCP-treated cells were normalized for variance in total IgM levels, and then subjected to analysis of IgM reactivity to YCP. To perform this, microplates pre-treated with poly-L-lysine were coated with 1 µg/well of YCP in carbonate buffer for 2 h at 37°C. The plates were washed three times and blocked for 1 h at 37°C with 1% BSA in PBS containing 0.05% Tween-20. After washes, culture supernatants diluted to 1/10, unless otherwise stated, were added to the plates and incubated for 2 h at 37°C. Bound IgM were detected using rat anti-mouse IgM and peroxidase-conjugated anti-rat IgG, followed by incubation with o-phenylenediamine substrate solution. Absorbance was measured at 492 nm with a reference of 630 nm.

### Fluoresceinamine labeling of YCP

The polysaccharide YCP was conjugated to FLA using the CDAP-activation method as previously described with slight modifications [45]. In brief, 10 mg of CDAP was added into an aqueous solution containing 30 mg of YCP with gentle stirring and maintained at pH 9.0 for 2.5 min. The CDAP-activated YCP were then mixed with 2 mg of FLA (pH adjusted to 8.0) and incubated at room temperature overnight. Fluoresceinamine-labeled YCP (fl-YCP) was separated from the excess free FLA with an Amicon Ultra-15 centrifugal filter unit (Millipore, Billerica, MA, USA). The FLA and YCP amounts in fl-YCP were respectively quantified by measuring absorbance at 440 nm and phenol-sulfuric acid assay [46]. Results for FLA labeling expressed as mole of FLA per mole of YCP were 52.0±3.8.

### Specific binding and competition assay

B cells were spotted on slides and stained with fl-YCP or FLA for 1 h on ice. Cells were then washed three times with ice-cold PBS containing 1% BSA, and photographed under a confocal laser scanning microscope (Olympus, Tokyo, Japan). In another experiment, aliquots of B cell suspensions at a density of 1×10^6^ cells/ml in PBS containing 1% BSA were incubated with fl-YCP at serial concentrations (20–400 nM) for 1 h. After three washes, cells were examined on a FACSCalibur flow cytometer (BD Biosciences, San Jose, CA, USA) with a 488 nm laser excitation and a 530 nm emission filter. Data were acquired from a minimum of 100 000 cells and analyzed using the FlowJo program (FreeStar, Ashland, OR, USA). To perform the competition assay, B cells were incubated with a mixture of 100 nM fl-YCP and unlabeled YCP (200–3 200 nM) for 1 h. The binding was then analyzed by flow cytometry as aforementioned.

### Antibody blocking and signaling inhibition assay

B cells were pretreated with anti-TLR2, anti-TLR4 or respective isotype controls (20 µg/ml) at 37°C for 2 h, or with SB203580, U0126, SP600125 and PDTC (20 µM) at 37°C for 30 min prior to addition of YCP (80 nM). Cell proliferation and total IgM production in the culture supernatants were determined 48 h later using MTT assay and ELISA, respectively.

### Western blot analysis

B cells were treated with medium or YCP (20, 100, 200 nM) as described above. After incubation for 30 min, cells were collected for extraction of cytoplasmic proteins using RIPA lysis buffer supplemented with protease and phosphatase inhibitor cocktail. Equal amount of proteins were resolved on 12% SDS-polyacrylamide gel, electro-transferred onto a BioTrace NT nitrocellulose membrane (Pall, Ann Arbor, MI, USA) and then incubated in TBST buffer (50 mM Tris, pH 7.6, 150 mM NaCl, 0.05% Tween-20) containing 3% BSA at 37°C for 2 h. The membrane was subsequently incubated with mAbs against p38, ERK, JNK, phosphor-p38, phosphor-ERK, phosphor-JNK, phosphor-IκB-α or β-actin at 4°C overnight, followed by incubation with the corresponding secondary antibodies conjugated with horseradish peroxidase at 37°C for 1 h. The protein bands were finally visualized using an enhanced chemiluminescence (ECL) system.

### Electrophoretic mobility shift assay (EMSA)

Nuclear extracts from medium- or YCP-treated B cells were prepared according to a published protocol [47], and assayed for NF-κB binding activity using the biotin-labeled double-stranded oligonucleotide containing the consensus binding sequence for NF-κB (5′-AGT TGA GGG GAC TTT CCC AGG C-3′). EMSA was performed by the Chemiluminescent EMSA kit following the manufacturer's instructions. Briefly, the biotin-labeled probe was incubated with the nuclear extracts in binding buffer for 20 min at room temperature, and then electrophoresed on a 6% non-denaturing polyacrylamide gel in 0.5× Tris-borate-EDTA buffer. After transferred and cross-linked into a Biodyne B nylon membrane (Pall, Ann Arbor, MI, USA), the biotin-labeled probes were subjected to chemiluminescence band-detection.

### Statistical analysis

The Prism 5.0 program (GraphPad Software, La Jolla, CA, USA) was used for statistical analysis. Mann–Whitney U test, or Kruskal–Wallis test followed by Dunn's post-hoc test were performed to determine significant differences where appropriate.

## Supporting Information

Figure S1 **The polysaccharide YCP is pure and highly homogeneous.** **A** and **B:** YCP was analyzed for its purity (A) and homogeneity (B) by high performance gel permeation chromatography. **C:** UV-scanning spectrum of YCP. **D:** Element analysis of YCP.
(TIF)
