# Supplementary figures and images for: Toll-Like Receptor 2 and Toll-Like Receptor 4-Dependent Activation of B Cells by a Polysaccharide from Marine Fungus Phoma herbarum YS4108
Source: PLoS One. 2013 Mar 29;8(3):e60781. doi: 10.1371/journal.pone.0060781 (PMC3612108; doi:10.1371/journal.pone.0060781)

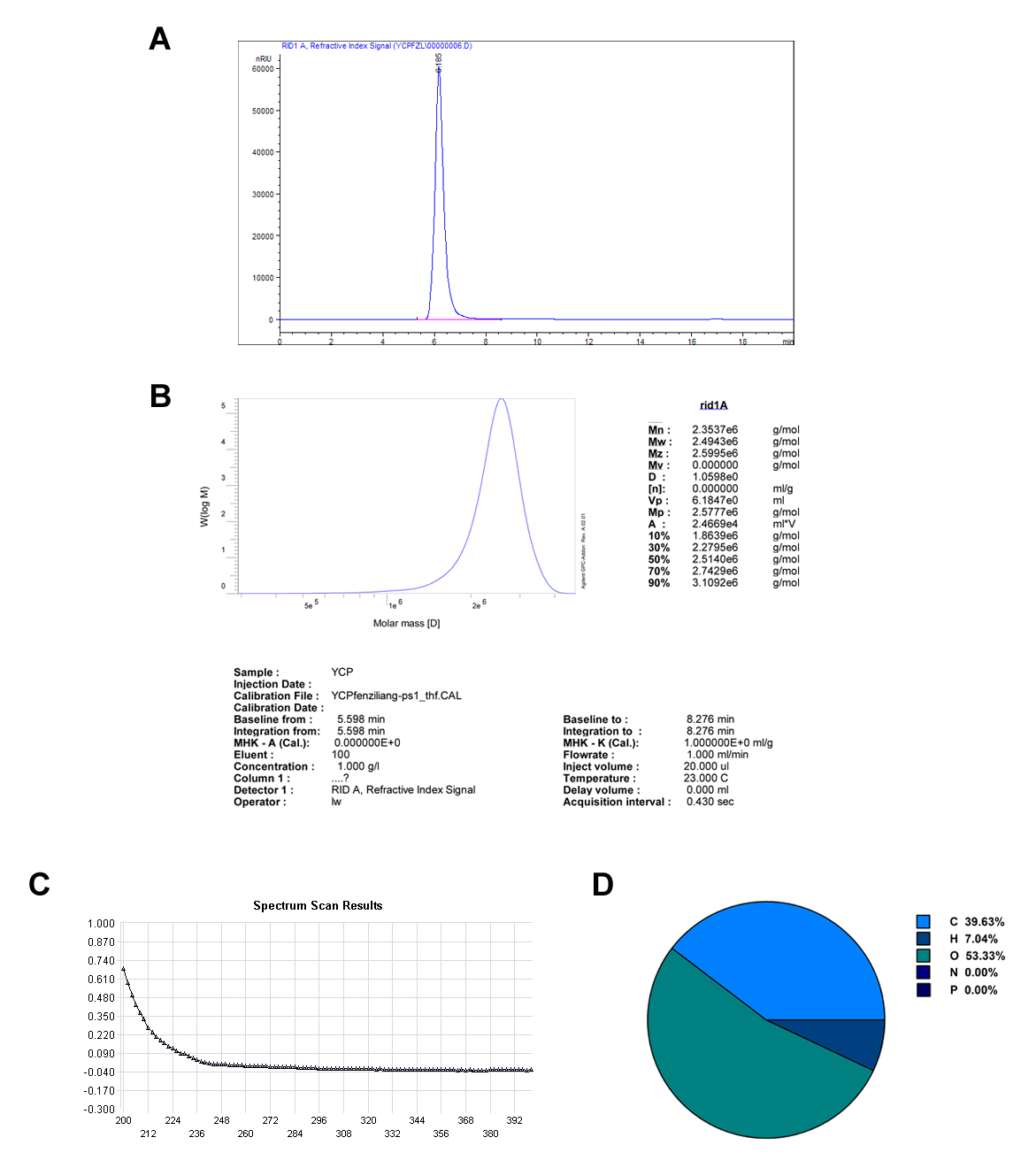

Supplement: Figure S1 — The polysaccharide YCP is pure and highly homogeneous. A and B: YCP was analyzed for its purity (A) and homogeneity (B) by high performance gel permeation chromatography. C: UV-scanning spectrum of YCP. D: Element analysis of YCP. (TIF) [file pone.0060781.s001.tif]
